# Successful use of immunoadsorption therapy in advanced dilated cardiomyopathy: A case report

**DOI:** 10.1097/MD.0000000000047080

**Published:** 2026-01-09

**Authors:** Rendan Zhang, Lvwen Ning, Ying Liu, Zhixiong Tan, Lu Kong, Xiaoqing Quan, Xiehui Chen

**Affiliations:** a Department of Geriatrics, Shenzhen Longhua District Central Hospital, Shenzhen, Guang Dong, P.R. China; b Shenzhen Longhua Key Laboratory of Personalized Precision Treatment for Elderly Coronary Heart Disease, Shenzhen, Guangdong, P.R. China.

**Keywords:** advanced DCM, dilated cardiomyopathy, immunoadsorption therapy

## Abstract

**Rationale:**

Dilated cardiomyopathy (DCM) is a major cause of chronic heart failure, and some patients continue to deteriorate despite guideline-directed medical therapy. Immunoadsorption (IA), which removes pathogenic autoantibodies, has been proposed as a potential adjunctive therapy, but its clinical use remains limited. This case illustrates the potential benefits of IA in advanced, treatment-refractory DCM.

**Patient concerns:**

A 53-year-old woman with a 9-year history of DCM presented with recurrent chest tightness and dyspnea. Despite long-term guideline-directed medical therapy, her symptoms progressively worsened.

**Diagnoses:**

The patient had chronic DCM initially diagnosed in the peripartum period, with a left ventricular ejection fraction (LVEF) of 11% at onset. Before IA, echocardiography demonstrated severe systolic dysfunction (LVEF 22%–24%) with significant mitral regurgitation, and laboratory evaluation showed markedly elevated pro-BNP (4454 pg/mL).

**Interventions:**

The patient underwent two sessions of immunoadsorption in September 2024, followed by high-dose intravenous immunoglobulin (IVIG) as per standard protocols.

**Outcomes:**

Following treatment, her symptoms improved, pro-BNP decreased to 2109 pg/mL, and repeat echocardiography showed an increase in LVEF to 29.2%.

**Lessons:**

This case suggests that immunoadsorption may offer clinical benefit in patients with advanced, refractory DCM. While promising, IA remains experimental and requires further investigation in larger, controlled studies.

## 1. Introduction

Dilated cardiomyopathy (DCM) is characterized by impaired myocardial contractility and ventricular dilation and is a major cause of heart failure worldwide.^[1,2]^ Clinically, patients present with a wide range of symptoms, including progressive dyspnea, orthopnea, ankle swelling, arrhythmias, thromboembolic events, and sudden cardiac death.^[3,4]^ Although pharmacotherapy, cardiac resynchronization therapy, implantable cardioverter-defibrillators, and heart transplantation are available,^[5]^ a subset of patients progress to refractory heart failure with poor outcomes.^[6,7]^

When secondary causes such as hypertension, coronary artery disease, and valvular disease are excluded, DCM is often linked to genetic predisposition, viral myocarditis, and autoimmune mechanisms.^[8–10]^ Among various autoantibodies, the anti-β₁-adrenergic receptor (anti-β₁-AR) antibody is the most extensively studied and best characterized. This antibody primarily recognizes the second extracellular loop of the β₁ receptor and exhibits agonist-like activity similar to endogenous ligands. Persistent receptor stimulation induces excessive activation of the cAMP signaling pathway, calcium overload, and mitochondrial dysfunction, ultimately leading to cardiomyocyte apoptosis and ventricular remodeling.^[11]^ Iwata et al demonstrated that anti-β₁-AR antibody levels are markedly elevated in patients with DCM and significantly correlate with the degree of left ventricular systolic impairment.^[12]^ Furthermore, removal of these antibodies via immunoadsorption has been associated with improvements in left ventricular ejection fraction (LVEF), suggesting a potential pathogenic role. In addition to β₁-receptor-related antibodies, anti-M₂ muscarinic receptor (anti-M₂) antibodies have been detected in a subset of DCM patients and are closely linked to arrhythmogenesis, possibly by disrupting parasympathetic regulation of cardiac rhythm.^[13]^ Other reported cardiac autoantibodies include anti-myosin and anti-cardiac troponin I (anti-cTnI) antibodies, both of which may directly alter cardiomyocyte ion channel function, promote calcium dysregulation, and contribute to contractile dysfunction. Immunoadsorption (IA) therapy, which selectively removes circulating immunoglobulins and immune complexes, has shown promise in autoimmune conditions and in patients awaiting transplantation.^[14–17]^ Emerging data suggest IA may improve cardiac function in DCM by eliminating pathogenic antibodies.^[18–20]^ However, clinical evidence remains limited.

Here we report the case of a 53-year-old woman with long-standing DCM refractory to medical therapy who experienced symptomatic and functional improvement after IA therapy combined with high-dose immunoglobulin.

## 2. Case presentation

A 53-year-old woman with a 9-year history of DCM presented with recurrent chest tightness and progressive dyspnea. Her initial episode occurred in the peripartum period following a febrile illness, when echocardiography at an outside hospital revealed a LVEF of 11% and markedly elevated N-terminal pro-B-type natriuretic peptide (BNP). She was diagnosed with DCM and subsequently treated intermittently, with inconsistent medication adherence, with sacubitril-valsartan, amiodarone, trimetazidine, spironolactone, metoprolol, and traditional Chinese medicine, but her symptoms persisted.

On July 29, 2024, she was admitted to another hospital with worsening dyspnea. Echocardiography demonstrated diffuse left ventricular hypokinesis, severely reduced systolic function, severe mitral regurgitation, and an LVEF of 24%. After receiving symptomatic treatment, including heart failure management, oxygen therapy, diuretics, anti-heart failure medications, ventricular remodeling therapy, and heart rate control, her condition improved. Implantation of an ICD was recommended, but the patient declined and was discharged.

The patient was subsequently referred to our hospital and readmitted on August 27, 2024, with recurrent chest tightness and mild dyspnea, accompanied by a dry cough. She denied abdominal distension, anorexia, lower-limb edema, nausea, vomiting, paroxysmal nocturnal dyspnea, or palpitations. At admission, she reported only mild fatigue. Her past medical history was unremarkable for hypertension or type 2 diabetes. Coronary angiography performed previously at an outside hospital was normal. Myocardial biopsy was not performed.

On examination, she was alert and afebrile, with a temperature of 36.5 °C, heart rate 78 beats per minute, respiratory rate 20 breaths per minute, and blood pressure 99/68 mm Hg. Lung sounds were diminished bilaterally without rales. Cardiac examination revealed cardiomegaly with an irregular rhythm, occasional premature beats, and a grade 2/6 systolic blowing murmur at the mitral area, without additional heart sounds. The abdomen was soft and non-tender, with no hepatosplenomegaly, and there was no edema in the lower extremities.

## 3. Auxiliary examinations

On admission (August 30, 2024), laboratory tests revealed a white blood cell count of 4.28 × 10^9^/L, hemoglobin 138 g/L, creatinine 77 µmol/L, potassium 4.03 mmol/L, total cholesterol 4.34 mmol/L, and low-density lipoprotein 2.71 mmol/L. Liver function tests showed ALT 14 U/L and AST 19 U/L, with albumin 43.4 g/L. Troponin was negative, D-dimer was 0.29 µg/mL, and thyroid function was normal. High-sensitivity C-reactive protein, rheumatoid factor, anti O, immunoglobulin levels (IgA, IgG, IgM), complement factors (C3, C4), systemic vasculitis markers, anti-cyclic citrullinated peptide, lupus screening, rheumatic panel, anti-histone antibodies, and anticardiolipin antibodies were all within normal ranges.

Serial measurements of N-terminal pro-BNP demonstrated persistently elevated values (see Table 1): 4436 pg/mL on July 29, 2024, 4454 pg/mL on August 27, 2024, 4128 pg/mL on September 6, decreasing to 2138 pg/mL on September 15, and 2109 pg/mL at follow-up on October 15, 2024, following immunoadsorption therapy on September 9 and 11. ICD implantation was again recommended during follow-up, but the patient continued to decline device therapy.

**Table 1:** Serial NT-pro-BNP, echocardiographic parameters, and clinical symptoms.

Date	NT-pro-BNP (pg/mL)	LVEDD (mm)	LVEF (%)	Clinical symptoms
2015 (initial)	Not available	Not available	11	Severe LV systolic dysfunction; postpartum onset
August 27, 2024	4454	77	22.1	Chest tightness, dyspnea; bilateral pleural effusion
September 14, 2024	2138	71	28	Marked improvement in chest tightness and dyspnea
October 15, 2024	2109	72	29.2	Chest tightness on exertion

NT-pro-BNP and LVEDD data from the initial 2015 diagnosis were not available.

LV = left ventricle/left ventricular, LVEDD = left ventricular end-diastolic diameter, NT-pro-BNP = N-terminal pro-B-type natriuretic peptide, LVEF = left ventricular ejection fraction.

**Figure 1.**
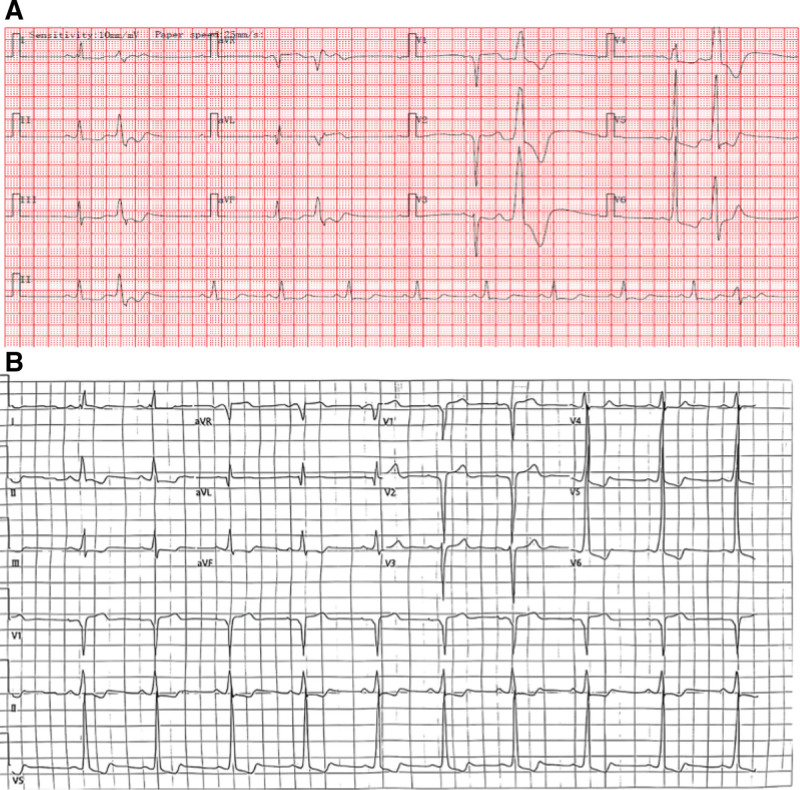
Electrocardiogram and Holter monitoring findings. (A) Baseline electrocardiogram on August 30, 2024, demonstrating intraventricular conduction block, poor R-wave progression, and frequent ventricular premature beats. (B) Follow-up tracing on October 15, 2024, showing improved rhythm stability after immunoadsorption therapy.

Electrocardiography and Holter monitoring (Fig. 1) demonstrated intraventricular conduction block, poor R-wave progression, frequent ventricular premature beats, and short episodes of ventricular tachycardia. The baseline Electrocardiogram (ECG) obtained on August 30, 2024, and the follow-up tracing from October 15, 2024, are presented in Figure 1A and B, respectively.

**Figure 2.**
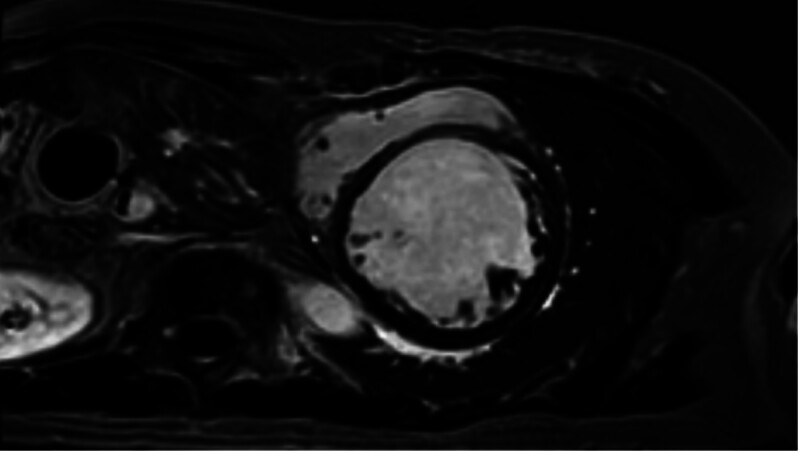
Cardiac MRI. Cardiac MRI performed on August 30, 2024, showing global cardiac enlargement, diffuse thinning of the left ventricular wall, and linear mid-myocardial fibrosis in the interventricular septum. The LVEF was estimated at 24%. LVEF = left ventricular ejection fraction, MRI = magnetic resonance imaging.

**Figure 3.**
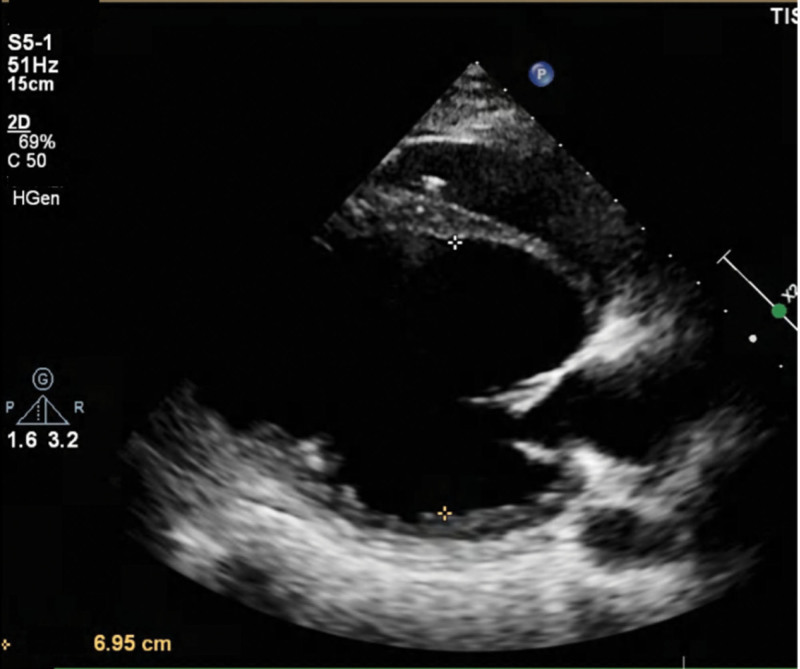
Transthoracic echocardiography. Follow-up echocardiograms demonstrated progressive improvement in cardiac function after immunoadsorption therapy. On September 14, 2024, the left atrium measured 38 mm, the left ventricle 71 mm, with moderate mitral regurgitation and an LVEF of 28%. At the external hospital follow-up on October 15, 2024, the LVEF further improved to 29.2%. LVEF = left ventricular ejection fraction.

Echocardiography and cardiac magnetic resonance imaging demonstrated significant ventricular dilation and impaired function. Echocardiography on August 27 showed an enlarged left atrium (38 mm) and left ventricle (77 mm), moderate-to-severe mitral regurgitation, mild tricuspid regurgitation, global and segmental wall motion abnormalities, and an LVEF of 22.1%. Cardiac magnetic resonance imaging on August 30 confirmed global cardiac enlargement, diffuse thinning of the left ventricular wall, and linear mid-myocardial fibrosis of the interventricular septum, with an EF of 24% (Fig. 2). Follow-up echocardiography on September 14 revealed a left atrium of 38 mm, left ventricle of 71 mm, moderate mitral regurgitation, and LVEF of 28%. At the external hospital follow-up on October 15, echocardiography showed an LVEF of 29.2% (Fig. 3 and Table 1).

## 4. Diagnostic process

The diagnosis of DCM was established according to the *Chinese Guidelines for the Diagnosis and Treatment of Dilated Cardiomyopathy (2018*), based on echocardiographic evidence of significant ventricular dilation and systolic dysfunction, along with markedly elevated pro-BNP levels. Electrocardiography and Holter monitoring demonstrated intraventricular conduction block, poor R-wave progression, frequent ventricular premature beats, and brief episodes of ventricular tachycardia. The patient’s lowest documented LVEF was 11%, consistent with advanced dilated cardiomyopathy refractory to medical therapy. Following admission, symptomatic therapy combined with immunoadsorption resulted in marked clinical improvement, reflected by a decline in BNP levels and partial recovery of LVEF, suggesting therapeutic benefit from this approach in advanced DCM.

## 5. Treatment plan

The patient was managed with a combination of pharmacologic and supportive therapies. She received Qili Qiangxin capsules (1.2 g orally, 3 times daily) for cardiac support, Shenqi Fuzheng injection (250 mL intravenous infusion, once daily) to enhance immune function, and fructose-1,6-diphosphate injection (50 g intravenous infusion, once daily) to support myocardial metabolism. Guideline-directed medical therapy included dapagliflozin (10 mg orally, once daily), finerenone (10 mg orally, once daily), sacubitril-valsartan (25 mg orally, twice daily), metoprolol (12.5 mg orally, twice daily), and trimetazidine (20 mg orally, 3 times daily). Rivaroxaban (15 mg orally, once daily) was administered for anticoagulation and thrombosis prevention, while Wenxin granules (5 g orally, 3 times daily) provided antiarrhythmic effects. Escitalopram (10 mg orally, once daily) was prescribed for anxiety management, along with diuretics and electrolyte regulation as needed. Immunoadsorption therapy was performed on September 9 and 11, 2024, followed by IVIG replacement.

## 6. Discussion and conclusion

This report describes a 53-year-old woman with long-standing DCM with severe systolic dysfunction who derived clinical benefit from immunoadsorption therapy combined with high-dose immunoglobulin. Despite years of guideline-directed pharmacological treatment, her symptoms remained inadequately controlled, and her ventricular function progressively declined. Following immunoadsorption, she demonstrated symptomatic relief, improved exercise tolerance, an increase in LVEF, and a substantial reduction in pro-BNP levels.

Nine years after her initial diagnosis, the patient’s condition had progressed to advanced dilated cardiomyopathy with severe systolic dysfunction, and her LVEF had fallen to as low as 11%. Although medical therapy temporarily improved her LVEF to 22.1%, she continued to experience dyspnea and arrhythmias. Prior studies suggest that up to 25% of acute DCM cases may show functional recovery, yet patients with prolonged symptoms and severely impaired baseline function often have poor outcomes.^[21]^ The natural course of untreated DCM is grim, with 1-year survival of approximately 70% to 75% and only 50% at 5 years.^[22]^ This underscores the urgent need for alternative therapies in refractory cases.

Accumulating evidence highlights the role of autoimmunity in the pathogenesis of DCM. A range of antimyocardial antibodies – including those directed against mitochondrial adenine nucleotide translocase, adrenergic and muscarinic receptors, myosin heavy chains, and L-type calcium channels – can impair myocardial signaling, disrupt contractility, and provoke inflammatory cascades. Complement activation further exacerbates myocardial injury and contributes to pathological remodeling.^[23]^

Immunoadsorption selectively removes these circulating autoantibodies, thereby attenuating immune-mediated myocardial damage.^[24–26]^ Clinical studies have demonstrated that this intervention can improve ventricular function, particularly in autoimmune-mediated cardiomyopathy.^[25,27,28]^ In our patient, LVEF increased from 24% to 29.2%, and BNP levels fell by more than 50%, consistent with both symptomatic and biochemical improvement. Importantly, no adverse events were observed, in line with prior reports supporting the safety and tolerability of this therapy.^[17,29]^

Although the patient showed symptomatic and echocardiographic improvement following IA, this treatment remains investigational and cannot replace established advanced heart failure therapies. Heart transplantation remained the definitive therapy for this patient but was declined, and ICD implantation was also refused, which influenced the decision-making toward IA as an exploratory adjunctive option.

This case highlights the potential utility of immunoadsorption as a personalized treatment option for patients with refractory DCM. Nevertheless, several limitations should be acknowledged. This is a single-patient observation and cannot be generalized to the broader DCM population. Autoantibody levels were not measured before or after therapy, which precludes direct mechanistic confirmation. Speckle-tracking echocardiography was not performed in this case. This modality provides sensitive assessment of myocardial fibrosis and may improve prognostic risk stratification in DCM patients, as demonstrated by recent studies.^[30]^ Furthermore, follow-up was relatively short, and the long-term durability of the therapeutic response remains uncertain. At present, IA for DCM is used only in selected centers and remains an investigational strategy without guideline endorsement. Current evidence is insufficient for routine clinical use, and IA should be considered exploratory and adjunctive rather than therapeutic. Future prospective studies are warranted to better define patient selection, long-term efficacy, and safety.

In conclusion, this case provides supportive evidence that immunoadsorption therapy may benefit patients with advanced DCM. By improving symptoms, cardiac function, and biomarker profiles, immunoadsorption offers a promising adjunctive approach in the management of advanced DCM. Personalized, immunologically targeted strategies such as this could represent an important step toward improving outcomes in this challenging patient population.

## Author contributions

**Conceptualization:** Rendan Zhang, Lvwen Ning, Xiehui Chen.

**Formal analysis:** Rendan Zhang, Lvwen Ning.

**Funding acquisition:** Xiehui Chen.

**Resources:** Ying Liu, Zhixiong Tan, Lu Kong, Xiaoqing Quan.

**Writing – original draft:** Rendan Zhang, Lvwen Ning.

**Writing – review & editing:** Rendan Zhang, Lvwen Ning, Ying Liu, Zhixiong Tan, Lu Kong, Xiaoqing Quan, Xiehui Chen.
